# N. Y. State Academy of Veterinary Science and Comparative Pathology

**Published:** 1885-07

**Authors:** 


### Proceedings Veterinary Medical Societies.

### N. Y. STATE ACADEMY OF VETERINARY SCIENCE AND COMPARATIVE PATHOLOGY.

The last meeting of the season was held at the Pierrepont House, Brooklyn, June 12th, Dr. H. E. Earl, President, in the chair, and Dr. Charles A. Meyer, Recording Secretary. The following members were present: Louis V. Plageman, James Hamill, Harry D. Gill, Peter Peters, William D. Middleton, Thomas Robertson, William H. Rohrs, John H. Dancer, John A. McLaughlin, William Hepburn, Charles B. Uibel, Daniel Schmidt and John T. Riley. Regrets were received from Rob't Laidlaw, M.R.C.V.S., of Albany; John Lindsay, D.V.S., of Huntington; J. S. Sutcliffe, V.S., of Port Jervis; Dr. J. Freeman, of N. Y., and Dr. Peter C. Juhl, of Staten Island. The medical profession was represented by some of its most prominent members.

The principal subject of the evening was "Glanders" and "Farcy," the dreaded scourge of the equine species.

Dr. McLaughlin, of Providence, R. I., gave a detailed account of a case under his supervision in a recent outbreak in his vicinity. He carefully followed the best known authors in the etiology, symptoms, diagnosis and results, and substantiated those by his own extended experience during his official connection with the State Board of Health in New Jersey, and during his practice in R. I. His conclusions were that although glanders in its incipiency could be suppressed, still it arose from the germ of glanders, which is a specific poison of unknown chemical composition and action, and always reproduces glanders and farcy, terminating fatally.

Dr. Charles A. Meyer, of N. Y., next spoke, and supported in many respects the views of Dr. McL., but was of opinion that glanders might also be *spontaneous*, and mentioned a case which occurred in his practice, in 1882,, in a stable where there were twelve horses, which, with the stable, were in a filthy condition, and had been so for years. The horses were improperly and irregularly fed and overworked. One of the animals developed glanders, and within a month the remainder were also affected. In four months they all died. At the same time the horses of the Central Park Carriage Service, which worked in the same harness and drank out of the same trough, never contracted the disease.

How are we to account for the above circumstance, if contagion is the only means of producing the disease?

Dr. John H. Dancer, of Orange, N. J., gave his views in accordance with those of Dr. McL., and related several cases he had observed during the recent outbreak in N. J.

Dr. L. V. Plageman, of Brooklyn, asked if any one present could furnish a positive diagnosis of glanders?

Dr. Meyer replied that Prof. Virchow has said that there was no actual physical diagnosis, but there was a microscopical diagnosis to the effect that spindle-shaped cells were found within the sub-maxillary lymphatic glands. This was doubted by the majority of the members, with all due respect to the leading pathologist, Virchow.

Dr. Plageman then said that *Inoculation* was a sure test, if properly applied, but the most decisive proof was to "cut up" an affected animal *after* he was *dead.* The most useful and scientific discovery would be to diagnose correctly the disease whilst the animal is still alive, therefore inoculation was the test he advanced, to be relied on, together with a peculiar, haggard, anxious expression, the peculiar induration of the sub-maxillary lymphatic glands, the nasal discharge (glutinous), the unthriftiness of the coat, invariably loss of appetite, and many other marked symptoms, according to its duration, age and surroundings.

The chairman, in rather a sarcastic strain, stated that science had arrived at such a fine point that veterinarians knew very litile of what is and what is not glanders.

Dr. James Hamill, of New York, objected to the terms glanders and farcy, but supposed they were used for want of a better, and said if we could obtain an authentic history of the glandered horse, we should find that, in the majority of cases or instances, first, he has been dull, off his feed and losing flesh, and his coat sometimes rough or staring. Also, that these appearances had for several weeks preceded, with more or less intensity, the characteristic symptoms of glanders, viz.: A continuous discharge from the nose, which discharge is sometimes so light as to be scarcely perceptible; at others to be very considerable and continue for weeks and months before the health and capabilities of the animal seemed to be injured. When pus appears in the discharge then another and very important characteristic appears, viz.: Swelling of the glands under the jaw. The lymphatic on one or both sides, more generally one side. This affection of the glands may progress until the entire glandular system succumbs to it. If the discharge be from one nostril the swelled gland will be found on that side, and there is a peculiarity about the swelled gland. The swelling at first may be large and diffused, but the surrounding swelling soon goes off and one or two small, distinct glands remain, and they are not in the centre of the maxillary space, but adhere closely to the jaw on the affected side. The membrane of the nose may materially guide our opinion at this stage. It will generally be of a dark leaden hue, or of some bluish shade. At any rate there will not be found the redness of inflammation nor the pink-red of health. Some spots of ulceration will probably appear on the membrane covering the cartilage of the nose—not simple sore spots or streaks of abrasion, and superficial, but small ulcers approaching to the circular form, with the edges abrupt and prominent or well defined—in which case there can be no doubt about its being glanders. At this stage the constitution will be evidently affected. The horse will lose flesh, the abdomen will be tucked up, the coat will be unthrifty and readily come off; occasionally a cough will be heard, the appetite impaired, the strength will fail, the discharge from the nose will grow more purulent, the ulcers in the nose become larger and more numerous. The air passages begin to be obstructed. A grating sound and sometimes choking noise will be heard at every act of breathing. The lungs are now diseased and filled with tubercles or ulcers and the horse at length dies, an emaciated and loathsome object. But as the early symptoms vary in a most puzzling degree, here our knowledge and judgment are most required.

Glanders have been confounded with strangles or distemper, with catarrh or common cold. It has oftener been confounded with catarrh than any other disease, and between those two especially we are called upon to decide. The discharge from catarrh, even when it is in the chronic form, does not feel so sticky as that of glanders; it does not form the crusty border of the nostrils as does that of glanders, and especially it does not form an upper border of the nostril, while we find that of glanders adhering all round the edge, and tending to mat the hairs together wherever it touches. Fever, more or less intense, accompanies catarrh, and the glands, if swollen, are movable, but at this stage of glanders there is no fever and the glands adhere to the jaw and are different in every way; the lymphatic glands inside the thighs become prominent and will suppurate. But in farcy all the absorbents of the skin seem to be affected at once, and the glands suppurate and discharge the same sticky matter as from the schneiderian membrane. Those suppurating openings are then known as farcy buds. The increase of these buds marks the progress of the disease, and although farcy itself often assumes different forms, the result of the disease is the same as that of glanders. It is only important in the early stages to diagnose between glanders and farcy, because only in the early stages is there any opportunity—with our present knowledge—of combating this disease, or these two forms of the same disease. In fact, some writers have claimed that there are three forms of this disease. Turner, for instance, has proposed a third kind, which he calls the insidious. This brings up another debatable point. Is glanders a specific poison? I know the greater number of authorities claim that glanders is a specific poison, and must be reproduced by glanders, and nearly all claim that it cannot be cured, and that no case of glanders ever was cured, and that the only proper treatment is to destroy every infected and suspected animal, and burn the carcass. To this line of action there are some objections, and, while I admit that every precaution must be taken to prevent other animals and man from being affected by it, I claim there is a time and a stage of the disease when it can be controlled, or probably, I should say, suppressed. If we were to stamp out glanders from the world entirely, would it not reappear under certain conditions? I, for one, claim that it would, just as syphilis would reappear if it were stamped out.

The history of glanders equina covers a period of 1500 years, and during that time, for every death of glanders in man produced by contagion from the horse, there is over one million of deaths by syphilis. Yet, do we ever hear our State authorities, or boards of health, recommend the stamping out *by death*, of all infected and suspected human beings? Therefore, why should we, under false impressions, destroy the horse when we can probably control or suppress his disease by the same measures as are used in the human disease above-mentioned, which is far more dangerous? Can it not, also, be shown that syphilis is the cause of tuberculosis in man, or rather, is there not some connecting link? I think there is.

Virchow has shown that syphilis exists in the "wild" hare of Europe; that the primary lesions are in the genital passages, and constitute in their microscopic character a connecting link between *tuberculosis* and *gummatous*

*growths*, so that one observer claims that this disease was not syphilis, but *tuberculosis* conveyed from animal to animal by sexual contact, and modified in the transit; and, to quote the words of a visitor present, Dr. Frederick Muro, which he learned from his preceptor, consumption was scrofula, scrofula was leprosy, and leprosy was syphilis modified in its passages from one to another.

It is a well-known fact that syphilis exists among the native horses in India, that the native mares have infected European stallions, and it has been asserted that horses affected in this manner have died of tuberculosis.

I now put the question to any member present: Has tuberculosis ever been cured? Now, we know the worst form of glanders is the tuberculous form. Is there any difference in tuberculosis, either in equine, bovine or human species? We find a connection between syphilis and tuberculosis in the human and a similar connection between glanders and tuberculosis in the horse, and there is a similarity of a diseased condition of the frontal and nasal bones in the *syphilitic human* and the *glandered horse*.

In support of Turner that glanders might occur spontaneously, otherwise, how could we account for well-authenticated sudden outbreaks of the disease, as in the British expedition to Quebourn in the year 1800, and in another, to the Crimea, in 1854.

The Chairman contended that it might originate by absorption from foul emanations—horses standing over ammoniacal gases, breathing a vitiated atmosphere and closely confined in hot and ill-ventilated buildings.

Dr. Plageman asserted that the disease was a specific poison which might be generated in the system by over-work, low diet and poisoned atmosphere.

Dr. McLauglin held that it was a disease produced by the virus of glanders, which must always be glanders or farcy, or why would it produce the same disease by inoculation, and asked what was its chemical composition; if of spontaneous origin?

Dr Peters disagreed with Dr. Hammill, for during his experience in the army, he found that horses not confined in close, vitiated atmospheres were subject to glanders, but admitted (without giving any reason) that the liability was greater during the time of war than in the time of peace.

Dr. Robertson quoted a case of syphilis in the eye of a horse, produced by inoculation from man, and it was subsequently admitted by the groom in charge that he had maliciously inoculated the eye of the animal.

Then the question was asked by several members was that specific poison syphilis? If so, the theory of Drs. Hammill and Moore would go to prove that a specific disease does not always produce the same disease when transmitted from one animal to another.

Dr. Hammill closed the debate by stating that the disease (glanders) originated in the pituitary membrane, its chemical composition being unknown, but upon that membrane the proof of glanders was looked for, and that any foul irritant or poison acting on this delicate membrane would probably receive its *specific character* by being absorbed.

The next meeting will be held at West Brighton, Richmond Co., N. Y., on September the 11th, 1885.

Dr. Plageman volunteered to read a paper on "Colic and Constipation."

Dr. Meyer one on "Mamitis in the Cow."

Dr. Reily, M.D., one on "Comparative Pathology of the above Diseases."

